# Financial burden among older, long‐term cancer survivors: Results from the LILAC study

**DOI:** 10.1002/cam4.1671

**Published:** 2018-07-17

**Authors:** Theresa A. Hastert, Gregory S. Young, Michael L. Pennell, Tasleem Padamsee, S. Yousuf Zafar, Cecilia DeGraffinreid, Michelle Naughton, Michael Simon, Electra D. Paskett

**Affiliations:** ^1^ Department of Oncology Wayne State University School of Medicine Detroit Michigan; ^2^ Population Studies and Disparities Research Program Karmanos Cancer Institute Detroit Michigan; ^3^ Department of Biomedical Informatics The Ohio State University College of Medicine Columbus Ohio; ^4^ Division of Biostatistics College of Public Health The Ohio State University Columbus Ohio; ^5^ Division of Health Services Management & Policy College of Public Health The Ohio State University Columbus Ohio; ^6^ The James Comprehensive Cancer Center The Ohio State University Columbus Ohio; ^7^ Department of Medicine Duke University School of Medicine Durham North Carolina; ^8^ Sanford School of Public Policy Duke University Durham North Carolina; ^9^ Division of Cancer Prevention and Control Department of Internal Medicine College of Medicine The Ohio State University Columbus Ohio

**Keywords:** cancer, cancer economics, cancer therapy, financial hardship, survivorship

## Abstract

**Background:**

Increasing attention is being paid to financial burdens of cancer survivorship, but little is known about the prevalence and predictors of these burdens in older, long‐term survivors.

**Methods:**

We used data from 6012 participants diagnosed with cancer since enrolling in the Women's Health Initiative, and who participated in the Life and Longevity After Cancer (LILAC) ancillary study to estimate prevalence and identify predictors of financial burden. We used logistic regression to identify sociodemographic, socioeconomic, health‐ and cancer‐related factors associated with financial burden and backward selection to build a final multivariable model.

**Results:**

Average age at LILAC participation was 79 and 9.2 years had elapsed since cancer diagnosis. Overall, 6% experienced some form of financial burden, including having an insurance company refuse a claim (2.6%), being denied loans or insurance due to cancer history (2.2%), or experiencing significant indebtedness (1.8%, including facing large debts or bills or declaring bankruptcy). Eight predictors remained associated (*P* < 0.05) with financial burden in the fully‐adjusted model: younger age, shorter time since diagnosis, African‐American race, household income <$20 000/year, modified Charlson comorbidity score ≥2, receipt of chemotherapy, regional stage at diagnosis, and no private health insurance. Education, cancer site, social support, receipt of radiation, and receipt of hormone therapy were not associated with financial burden. Predictors differed between types of financial burden experienced and age at diagnosis (<65 vs 65+).

**Conclusion:**

Cancer‐related financial burden was rare in this population of older, female long‐term cancer survivors. The identification of several socioeconomic, health‐related and demographic predictors of financial burden may suggest targets of intervention to reduce financial burdens.

**Precis:**

Financial burden was uncommon in older, female, long‐term survivors. Predictors of financial burden included age, race, income, comorbidities, time since diagnosis, stage, insurance, and receipt of chemotherapy.

## 1 INTRODUCTION

Many cancer survivors face serious financial burdens related to cancer.^1^ Although there is no single definition of financial burden, the concept of “financial toxicity” commonly includes both objective financial burdens experienced by cancer patients and survivors, and also subjective financial distress related to the cost of cancer treatment.^2^

Prevalence of financial burden varies depending on the population and the types of financial burden included;^3^ studies commonly report a prevalence of financial burden of 30%‐50%.^4^, ^5^ These burdens can include out‐of‐pocket costs and missed work days immediately surrounding diagnosis and treatment, but can also continue for years afterward.^6^, ^7^, ^8^ Cancer and cancer treatment can also lead to changes in survivors’ financial lifestyles, debt, and even bankruptcy.^9^, ^10^

Survivors who experience cancer‐related financial burdens experience poorer quality of life,^10^, ^11^, ^12^, ^13^, ^14^ and financial distress is a stronger predictor of quality of life than physical distress, symptom burden, depression, or anxiety.^15^ Financial burdens can also impact treatment decisions,^11^ which can negatively impact outcomes.^5^, ^16^, ^17^ Having health insurance does not prevent survivors from experiencing cancer‐related financial burdens,^18^—a cancer diagnosis can lead to changes in insurance status, increases in insurance premiums,^10^ and the inability to obtain or keep health or life insurance.^19^

Although research is increasingly highlighting the importance of financial burden in cancer survivorship, many prior studies have focused on small groups of survivors of an individual type of cancer or from one geographic region. Younger age is an established risk factor for financial burden among cancer survivors;^1^, ^10^, ^20^ but many older survivors also experience financial burden related to cancer, and the predictors of financial burden among older survivors are not well understood. The purpose of this paper is to estimate the prevalence and predictors of financial burdens due to cancer among older, long‐term survivors, who participated in the Life and Longevity After Cancer (LILAC) Study.

## 2 METHODS

### 2.1 Study participants

Women were eligible to participate in LILAC if they participated in the Women's Health Initiative (WHI) and the WHI Follow‐up Study, had no history of cancer other than nonmelanoma skin cancer when they enrolled in WHI, and were subsequently diagnosed with invasive breast, ovarian, endometrial, colorectal, lung, fallopian tube or peritoneum cancer, or with invasive melanoma, leukemia, or non‐Hodgkin's lymphoma.^21^ Beginning in 1992, WHI enrolled more than 160 000 postmenopausal women ages 50‐79 into a clinical trial or an observational study designed to investigate common causes of morbidity and mortality in this population. It has previously been described in detail.^22^, ^23^ WHI participants were recruited from areas around forty clinical centers, mostly at academic health centers, and recruitment took place in urban, suburban, and rural areas in 24 states and the District of Columbia.^24^ Minority participants were recruited in the same proportion as existed in the overall population, and clinics provided resources such as assistance with transportation, childcare, and parking reimbursement to overcome potential barriers to participation.^24^

The LILAC cancer survivorship cohort was designed with three study objectives: (a) to collect treatment and outcomes information from women diagnosed with cancer during their participation in WHI; (b) to supplement the WHI biorepository with tissue samples from select solid tumors; and (c) to test the ability of electronic administrative data to produce reliable treatment and recurrence data.^21^ A total of 30 306 women were diagnosed with incident cancers after enrolling in WHI. Of those, 9934 were invited to participate in LILAC after excluding women with cancers other than those selected for LILAC (N = 9522) and those who were otherwise ineligible (deceased, not in active WHI follow‐up, or with a history of cancer at LILAC baseline; N = 10 850). A total of 7634 women completed the baseline LILAC questionnaire (form 340, 2013‐2016), and 6012 are included here after excluding 1570 women who did not return the LILAC follow‐up questionnaire (form 370, 2014‐2016, which included the financial burden questions), 52 who did not answer the financial burden questions, and 3 diagnosed with in situ melanoma.

This study was conducted within the Belmont Report recognized ethical guidelines. Written informed consent was obtained from all participants and this study was approved by the Institutional Review Board of each institution.

### 2.2 Financial burden measures

On the LILAC follow‐up questionnaire, women were asked whether, since their cancer diagnosis, they had ever experienced any of the following (yes/no): denied health insurance, denied life insurance, health insurance company refused to pay a medical expense insurance claim, cancer treatment left them with large debts/bills to pay, trouble getting a mortgage or other loan because of their cancer history, declared bankruptcy because of their cancer. Women were counted as experiencing any financial burden if they answered “yes” to any of these items.

### 2.3 Predictors of financial burden

We obtained education, income, and race from the baseline WHI demographics dataset and cancer type, stage, and days from enrollment to diagnosis from WHI adjudicated outcomes, breast cancer outcome detail, and cancer outcome detail datasets from November 2016. Insurance at diagnosis, social support and self‐reported treatment for cancer were obtained from LILAC form 340.

We calculated a modified Charlson comorbidity index utilizing baseline and follow‐up WHI data.^25^ This modified scale differed from the standard 19‐item index in that several components (dementia, hemiplegia/paraplegia, AIDS, severe liver disease and diabetes with organ damage) were not included in WHI surveys or were exclusion criteria for enrollment. Where available, we used adjudicated outcomes to update baseline data (eg cancer diagnoses, myocardial infarction), otherwise we used self‐reported information (eg connective tissue disease). Some comorbidities assessed at baseline (eg ulcerative disease) were not included in follow‐up and were not updated. The LILAC‐associated cancer was not included in the score, but subsequent cancers contributed. Recurrence was assessed via self‐report from LILAC form 340. The range for this modified index was 0‐25 with higher scores denoting more comorbidity.

### 2.4 Statistical analysis

We defined four outcomes in this study based on the survey questions (a) insurance refused to pay a claim; (b) denied loans or insurance (including being denied health or life insurance, or having trouble getting loans because of cancer history); (c) indebtedness (including reporting that cancer treatment left a large debts or bills, or bankruptcy); and (d) an overall measure of any financial burden. Logistic regression models estimated associations between each predictor and each of these outcomes. Due to the high missingness among the predictors (18% of participants had a least one missing value) we performed a fully conditional specification multiple implementation procedure.^26^ Continuous variables were imputed using linear regression, dichotomous and ordinal variables using logistic regression, and nominal variables using a discriminant function. Variables significant at the *P* < 0.20 level or below for at least one of the outcomes were included in the imputation model. Responses to nine social support questions from LILAC form 370 were also used in the imputation model for the social support construct collected on the LILAC baseline survey. Forty imputed data sets were created using PROC MI, which follows the recommendation that the number of imputed data sets exceed the percentage of missing data.^27^

We used backward selection to build the final models. Each variable associated with any of the four financial burden outcomes (*P* < 0.20) in the single predictor models was included in the initial model for backward selection. At each step of the backward selection, potential predictors were eliminated based on the *P*‐value (*P* < 0.05 criterion) obtained by combining the estimates from 40 imputed datasets.^28^ Model estimates were combined using PROC MIANALYZE. All analyses were conducted using SAS 9.4 (SAS Institute, Inc., Cary, NC).

## 3 RESULTS

On average, at the time of the LILAC follow‐up participants were 79 years of age (range: 66‐98) and 9.2 years had elapsed since cancer diagnosis (Table 1). The majority (91.4%) were white, completed college (52.1%) and had some form of private health insurance at diagnosis (87.7%). Breast cancer accounted for more than half (58.8%) of the cancer diagnoses in this cohort, followed by colorectal (10.5%), endometrial (8.9%), melanoma (6.4%), and non‐Hodgkin's lymphoma (6.0%). Leukemia, lung, ovarian, peritoneum, and fallopian tube cancers each accounted for less than 5% of the cancer diagnoses. Most cancers (71.9%) were diagnosed at early stages, but this varied by site. More ovarian (45.9%), non‐Hodgkin lymphoma (50.6%), peritoneum (73.3%), and leukemia (99.2%) cases were diagnosed at distant than local or regional stages, while the majority of melanoma (94.3%), breast (78.1%), endometrial (85.6%), colorectal (58.4%), and lung (64.5%) cancer cases were diagnosed at local stages.

**Table 1:** Demographic, cancer, and treatment‐related characteristics of the analysis cohort

Variable	Total	
	N
	6012	%
Age at diagnosis (mean, SD)	69.8	7.5
Age at LILAC follow‐up (mean, SD)	79.0	5.9
Race		
White	5485	91.4
African‐American	242	4.0
Other	275	4.6
Educational attainment		
High school or below	881	14.7
Some college	1982	33.1
College graduate	1727	28.9
Graduate or professional degree	1390	23.2
Household income at baseline		
<$20 000	441	7.7
$20 000‐34 999	1143	20.0
$35 000‐49 999	1162	20.3
$50 000‐74 999	1399	24.5
$75 000+	1576	27.5
Insurance status at diagnosis		
Uninsured	39	0.7
Public only	341	6.2
Public/private combination	3479	62.9
Private insurance only	1371	24.8
VA with or without other insurance	300	5.4
Cancer site/type		
Breast	3535	58.8
Colorectal	630	10.5
Endometrial	534	8.9
Melanoma	387	6.4
Non‐Hodgkin's lymphoma	358	6.0
Lung	287	4.8
Leukemia	126	2.1
Ovarian	122	2.0
Fallopian tube	18	0.3
Peritoneum	2	0.03
Time since diagnosis		
<5y	1606	26.7
5+y	4405	73.3
Stage at diagnosis		
Local	4261	71.9
Regional	1229	20.7
Distant	438	7.4
Treatments received		
Chemotherapy	1802	30.2
Radiation	2861	47.9
Hormone therapy	2416	40.4
Modified Charlson score		
0	2777	46.2
1	1631	27.1
2+	1604	26.7
Social support construct		
Low (0‐65)	1714	31.2
Middle (66‐85)	1614	29.4
High (86‐100)	2159	39.3

LILAC, Life and Longevity After Cancer; SD, standard deviation; VA, United States Department of Veterans Affairs.

Financial burden was rare in this cohort (Table 2). Overall, 6% reported some form of financial burden. The most common form of financial burden was having an insurance company refuse a claim (2.6%); followed by being denied loans or insurance (2.2%), including 1.6% who reported being denied life insurance, 0.6% who were denied health insurance, and 0.1% who had trouble getting a mortgage or other loans; and indebtedness, including 1.7% who reported being left with large debts or bills due to treatment and 0.2% who declared bankruptcy because of cancer. Most of the women reporting financial burden reported only one form, with only 0.7% of the total study population reporting two or more.

**Table 2:** Prevalence of financial burden domains, individual financial burden measures and any financial burden

	N	%
Insurance refused claim	159	2.6
Denied loans or insurance	133	2.2
Denied health insurance	37	0.6
Denied life insurance	93	1.6
Trouble getting a mortgage or other loans because of cancer history	8	0.1
Indebtedness	105	1.8
Large debts or bills to pay due to cancer treatment	99	1.7
Had to declare bankruptcy because of cancer	12	0.2
Any financial burden (yes to any of the above questions)	358	6.0
Number of financial burden measures experienced:		
None	5654	94.1
1	316	5.3
2+	42	0.7

Responses are not mutually exclusive. The measure of being denied loans or insurance includes being denied health insurance, being denied life insurance, and reporting having trouble getting a mortgage or other loans. The measure of indebtedness includes reporting having large debts or bills and having to declare bankruptcy.

The association between each predictor and any financial burden is presented in Table 3. Age at LILAC participation was inversely associated with financial burden (OR per year: 0.93, 95% CI: 0.91, 0.94) and odds of experiencing any financial burden among women ages 65+ at diagnosis were 51% lower than among women diagnosed younger than 65 (OR: 0.49, 95% CI: 0.39, 0.61). African‐American women had 2.96 times the odds of reporting financial burden of white women (95% CI: 2.04, 4.29). Women with household incomes of $20 000 or more had significantly lower odds of financial burden compared to women with higher incomes (ORs of 0.48‐0.60). Women with a combination of public and private insurance at diagnosis had approximately half the odds of reporting any financial burden of those with only public insurance such as Medicare or Medicaid (OR: 0.44, 95% CI: 0.30, 0.65). Compared to women diagnosed with breast cancer, odds of financial burden were lower among women diagnosed with melanoma (OR: 0.52, 95% CI: 0.29, 0.94) and higher among those with leukemia (OR: 1.88, 95% CI: 1.04, 3.39). Financial burden was more common among women diagnosed with regional (OR: 1.69, 95% CI: 1.32, 2.15) or distant disease (OR: 1.74, 95% CI: 1.21, 2.50) than those diagnosed with local disease. The odds of financial burden among women who received any chemotherapy was nearly twice that of women who did not (OR: 1.97, 95% CI: 1.58, 2.44). Financial burden was more common among women with higher comorbidity burden (OR: 1.59, 95% CI: 1.24, 2.04 for modified Charlson score of 2+ vs 0), and was less common among women with the highest levels of social support compared with those with the lowest (OR: 0.74, 95% CI: 0.57, 0.97). Educational attainment, time since diagnosis, and receipt of radiation or hormone therapy were not associated with financial burden.

**Table 3:** Associations of demographic, clinical, and treatment‐related variables with financial burden

	Any burden		Any financial burden	*P*‐value
	N	%	OR (95% CI)	
Age at LILAC participation [mean (SD)]	76.6	(5.4)	0.93 (0.91, 0.94)	<0.001
Age at diagnosis				
<65	138	9.4	1.00	<0.001
65+	220	4.8	0.49 (0.39, 0.61)	
Race/ethnicity				
White	306	5.6	1.00	<0.001
African‐American	36	14.9	2.96 (2.04, 4.29)	
Other	16	5.8	1.05 (0.62, 1.75)	
Educational attainment				
High school graduate or below	43	4.9	1.00	0.16
Some college	136	6.9	1.44 (1.01, 2.04)	
College graduate	96	5.6	1.15 (0.79, 1.66)	
Graduate or professional degree	83	6.0	1.24 (0.85, 1.81)	
Household income at baseline				
<$20 000	45	10.2	1.00	0.003
$20 000‐34 999	61	5.3	0.50 (0.33, 0.74)	
$35 000‐49 999	74	6.4	0.60 (0.41, 0.88)	
$50 000‐74 999	82	5.9	0.56 (0.37, 0.80)	
$75 000+	82	5.2	0.48 (0.33, 0.71)	
Social support				
Low (0‐65)	118	6.9	1.00	0.076
Medium (66‐85)	91	5.6	0.81 (0.61, 1.07)	
High (86‐100)	112	5.2	0.74 (0.57, 0.97)	
Insurance status at diagnosis				
Uninsured	7	17.9	1.98 (0.81, 4.82)	<0.001
Public only	34	10.0	1.00	
Private insurance	101	7.4	0.72 (0.48, 1.08)	
Public/private combination	163	4.7	0.44 (0.30, 0.65)	
VA with or without other insurance	7	2.3	0.22 (0.09, 0.49)	
Cancer site/type				
Breast	204	5.8	1.00	0.057
Colorectal	44	7.0	1.23 (0.87, 1.72)	
Endometrial	33	6.2	1.08 (0.74, 1.57)	
Melanoma	12	3.1	0.52 (0.29, 0.94)	
Non‐Hodgkin's lymphoma	17	4.7	0.81 (0.49, 1.35)	
Lung	20	7.0	1.22 (0.76, 1.97)	
Leukemia	13	10.3	1.88 (1.04, 3.39)	
Ovarian	11	9.0	1.62 (0.86, 3.05)	
Fallopian tube	2	11.1	2.04 (0.47, 8.94)	
Peritoneum	2	13.3	2.51 (0.56, 11.21)	
Time since diagnosis				
<5 y	102	6.4	1.00	0.43
5+ y	256	5.8	0.92 (0.72, 1.15)	
Stage at diagnosis				
Local	215	5.0	1.00	<0.001
Regional	101	8.2	1.69 (1.32, 2.15)	
Distant	37	8.4	1.74 (1.21, 2.50)	
Treatment (vs none)				
Any chemotherapy	160	8.9	1.97 (1.58, 2.44)	<0.001
Any radiation	180	6.3	1.12 (0.91, 1.39)	0.28
Any hormone therapy	150	6.2	1.07 (0.86, 1.32)	0.56
Modified Charlson score				
0	140	5.0	1.00	0.001
1	93	5.7	1.14 (0.87, 1.49)	
2+	125	7.8	1.59 (1.24, 2.04)	

CI, confidence interval; LILAC, Life and Longevity After Cancer; OR, odds ratio; SD, standard deviation; VA, United States Department of Veterans Affairs

Eight predictors remained associated (*P* < 0.05) with higher odds of any financial burden following backward selection model (Table 4): younger age at time of LILAC baseline, receipt of chemotherapy, African‐American race, household income less than $20 000 per year, comorbid conditions, low social support, regional disease, and having only public insurance.

**Table 4:** Multivariable model of predictors of any financial burden

	Any financial burden	*P*
	OR (95% CI)	
Age at LILAC participation (per year)	0.93 (0.90, 0.95)	<0.001
Time since diagnosis (per year)	0.92 (0.89, 0.95)	0.002
Chemotherapy		
No	1.00	0.005
Yes	1.45 (1.12, 1.88)	
Race/ethnicity		
White	1.00	0.001
African‐American	2.06 (1.39, 3.06)	
Other	0.95 (0.56, 1.61)	
Household income at baseline		
<$20 000	1.00	0.009
$20 000‐34 999	0.62 (0.40, 0.94)	
$35 000‐49 999	0.68 (0.45, 1.03)	
$50 000‐74 999	0.56 (0.37, 0.84)	
$75 000+	0.48 (0.31, 0.72)	
Modified Charlson score		
0	1.00	0.002
1	1.12 (0.85, 1.47)	
2+	1.59 (1.23, 2.06)	
Stage at diagnosis		
Localized	1.00	0.048
Regional	1.40 (1.05, 1.85)	
Distant	1.38 (0.93, 2.07)	
Insurance status at diagnosis		
Public only	1.00	<0.001
None	2.28 (0.93, 5.62)	
Private only	0.62 (0.40, 0.97)	
VA	0.26 (0.12, 0.60)	
Public and private	0.53 (0.35, 0.79)	

CI, confidence interval; LILAC, Life and Longevity After Cancer; OR, odds ratio; VA, United States Department of Veterans Affairs

Predictors differed by type of financial burden (Table 5). Only younger age at LILAC participation, receipt of chemotherapy, and the presence of two or more comorbid conditions were associated with insurance refusing to pay a claim. Younger age at diagnosis, shorter time since diagnosis, and lower household income at baseline were associated with reporting being denied loans or insurance due to cancer status. Several factors remained associated with indebtedness (reporting large debts or bills due to cancer or declaring bankruptcy), including younger age at diagnosis, shorter time since diagnosis, African‐American (vs white) race, receipt of any chemotherapy, lower household income, the presence of two or more comorbid conditions, and having only public health insurance at diagnosis (vs only private insurance, VA, or a combination of public and private insurance).

**Table 5:** Multivariable model of predictors of individual financial burden domains (insurance refused claim, denied loans or insurance, indebtedness)

	Insurance refused claim	*P*	Denied loans or insurance	*P*	Indebtedness	*P*
	OR (95% CI)		OR (95% CI)		OR (95% CI)	
Age at diagnosis (per year)			0.88 (0.85, 0.91)	<0.001	0.95 (0.91, 0.99)	0.009
Age at LILAC participation (per year)	0.95 (0.93, 0.98)	0.001				
Time since diagnosis (per year)			0.94 (0.89, 0.99)	0.013	0.87 (0.82, 0.92)	<0.001
Race/ethnicity						
White					1.00	<0.001
African‐American					3.23 (1.82, 5.74)	
Other					2.03 (0.98, 4.19)	
Chemotherapy						
No	1.00	0.007			1.00	<0.001
Yes	1.57 (1.13, 2.16)				3.23 (2.14, 4.88)	
Household income at baseline						
<$20 000			1.00	0.019	1.00	0.003
$20 000‐34 999			0.49 (0.25, 0.95)		0.77 (0.40, 1.48)	
$35 000‐49 999			0.47 (0.25, 0.90)		0.66 (0.34, 1.29)	
$50 000‐74 999			0.52 (0.29, 0.96)		0.45 (0.23, 0.90)	
$75 000+			0.33 (0.17, 0.62)		0.24 (0.11, 0.52)	
Modified Charlson score						
0	1.00	0.042			1.00	0.007
1	1.16 (0.77, 1.75)				1.37 (0.82, 2.28)	
2+	1.90 (1.32, 2.74)				2.15 (1.33, 3.48)	
Insurance status at diagnosis						
Public only					1.00	<0.001
None					2.73 (0.69, 10.84)	
Private only					0.45 (0.23, 0.90)	
VA					0.07 (0.01, 0.53)	
Public and private					0.29 (0.16, 0.52)	

CI, confidence interval; LILAC, Life and Longevity After Cancer; OR, odds ratio; VA, United States Department of Veterans Affairs

Predictors of any financial burden also differed by age at diagnosis (Table 6). Only receipt of chemotherapy and health insurance at diagnosis (private only or combination of public and private vs public only) were associated with any financial burden among survivors younger than 65 at diagnosis. Among those ages 65 and older at diagnosis, older age at diagnosis, younger age at LILAC participation, receipt of chemotherapy, African‐American race, presence of two or more comorbid conditions, only public health insurance (vs VA or a combination of public and private insurance), and leukemia and colorectal cancer (vs breast cancer) were associated with experiencing any financial burden.

**Table 6:** Multivariable model of predictors of any financial burden, stratified by age at cancer diagnosis (<65, 65+)

	Age <65 at diagnosis		Age 65+ at diagnosis	
	OR (95% CI)	*P*	OR (95% CI)	*P*
Age at diagnosis (per year)			1.06 (1.02, 1.10)	0.007
Age at LILAC participation (per year)			0.90 (0.87, 0.94)	<0.001
Chemotherapy				
No	1.00	0.040	1.00	<0.001
Yes	1.45 (1.02, 2.07)		1.89 (1.40, 2.56)	
Race/ethnicity				
White			1.00	<0.001
African‐American			2.58 (1.57, 4.24)	
Other			0.88 (0.44, 1.76)	
Modified Charlson score				
0			1.00	<0.001
1			1.23 (0.87, 1.74)	
2+			1.89 (1.35, 2.64)	
Insurance status at diagnosis				
Public only	1.00	0.005	1.00	0.003
None	1.32 (0.45, 3.87)		2.52 (0.36, 17.79)	
Private only	0.38 (0.19, 0.76)		0.60 (0.33, 1.11)	
VA	0.31 (0.08, 1.18)		0.19 (0.06, 0.56)	
Public and private	0.40 (0.19, 0.85)		0.49 (0.31, 0.78)	
Cancer site/type				
Breast			1.00	0.031
Colorectal			1.61 (1.08, 2.42)	
Endometrial			1.19 (0.70, 2.00)	
Melanoma			0.66 (0.30, 1.45)	
Non‐Hodgkin's lymphoma			0.79 (0.44, 1.44)	
Lung			1.50 (0.89, 2.55)	
Leukemia			2.56 (1.28, 5.13)	
Ovarian			0.93 (0.40, 2.13)	
Fallopian tube			2.90 (0.60, 13.93)	

CI, confidence interval; LILAC, Life and Longevity After Cancer; OR, odds ratio; VA, United States Department of Veterans Affairs

## 4 DISCUSSION

With an observed prevalence of 6%, financial burden as measured by domains of insurance refusing to pay a claim, being denied loans or insurance, or indebtedness due to cancer, was rare in this population of older, female, long‐term cancer survivors. This is lower than many previous estimates of financial burden among cancer survivors; however, differences in the types of financial burden considered, the age at diagnosis and the length of survival of LILAC participants could contribute to these different findings.

There is no one consistent measure of financial burden among cancer survivors; however, several previous studies have included cancer‐related debt and bankruptcy. In one review, 12%‐62% of survivors reported being in debt due to cancer treatment.^3^ In a national sample, 7.7% of survivors not in active treatment reported having cancer‐related debt,^29^ and in another population 12% of breast cancer survivors reported still having debt 4 years after diagnosis.^6^ Among LILAC participants, 1.7% reported being left with large debts or bills to pay, much lower than estimates in previous work. Previous work suggests that bankruptcy is rare (1.5%‐3.1%) among cancer survivors,^10^, ^29^, ^30^ but our observed prevalence of bankruptcy following cancer diagnosis (0.2%) is much lower than in previous studies. Differences in question wording, survivor populations studied, and in time since diagnosis could account for the variation in these results.

The LILAC questionnaire includes measures of financial difficulties not commonly reported in other work on the financial consequences of cancer. The most common form of financial burden reported by LILAC participants was having an insurance company refuse to pay a medical insurance claim. It is not clear from the previous literature how common this is among other populations of cancer survivors. Among women with stage 0‐II breast cancer, Meneses et al reported that 16% experienced an increase in insurance premiums and 4.6% exceeded the benefits covered by their insurance companies,^10^ but these measures are not directly comparable to those in LILAC, and this population of survivors included women as young as 21. It is also not clear that the experience of having an insurance company refuse to pay a claim necessarily led to burden for the women who reported it. The majority of women in LILAC had more than one form of insurance and where one might have refused a claim, another might have paid it, or the woman may have been able to pay out of pocket for the treatment without experiencing financial distress.

Very few LILAC participants reported being denied health (0.6%) or life insurance (1.6%). The majority of LILAC participants were at least 65 years of age at the time of cancer diagnosis, meaning they would have been eligible for Medicare and probably less likely to be denied health insurance. Little comparable information exists in the literature; however, in a population of patients who underwent intensive treatment for multiple myeloma, Goodwin et al reported that 29% changed or lost insurance coverage, including 10% who were unable to obtain replacement insurance, and that although 70% had life insurance when they began treatment, 8% of those no longer had the same life insurance policy at study entry an average of 5 years after diagnosis.^19^

Women in LILAC were diagnosed with cancer an average of 9 years before answering the questions on financial burden. This would lead to survival bias if the prevalence of financial burden differed by whether women survived long enough to participate in LILAC. If financial burden was more common among women who died before LILAC recruitment, our observed prevalence of financial burden would be low. Additionally, longer time since diagnosis has been found to be inversely associated with financial burden among cancer survivors.^31^, ^32^ Recall bias could influence the results if women with the most severe financial burden remembered and reported those outcomes while survivors who were less dramatically impacted did not. There is also the possibility of survival bias if fewer women who survived and remained healthy enough to enroll in LILAC experienced financial burden than those who did not.

All LILAC participants were recruited from women who were part of the WHI, a long‐term prevention trial and observational study.^24^ Although the WHI attempted to remove barriers to participation, participants had to attend a clinic visit and agree to long‐term study participation before enrollment, requirements that were more easily met by women with financial resources who are also less likely to report financial burdens related to caner. Further, financial burden is less common among white than minority cancer survivors. WHI recruited minority participants in proportion to their presence in the general population (~18% at WHI enrollment); however, nonwhite women made up a smaller proportion of LILAC participants^21^ and account for only 8.6% of the women included here. Including fewer survivors from groups that most commonly report financial hardship would result in observed financial burden prevalence that is lower than in the general population.

Even among this population of older women, age was inversely associated with reporting any financial burden, consistent with previous work. Younger age has been positively associated with treatment‐related financial burden among survivors of colorectal cancer,^20^ higher perceived financial distress,^32^, ^33^ and greater likelihood of going into debt or filing for bankruptcy.^9^, ^30^ Little previous work has focused specifically on financial burden in older cancer survivors. Wiltshire et al reported that odds of medical debt were more than twice as high among older African‐American compared with older white survivors, similar to our findings.^34^ Davidoff et al reported that out‐of‐pocket medical costs were higher among Medicare beneficiaries with cancer than without, and that this higher burden was at least partially explained by the presence of comorbidities and lack of supplemental insurance.^35^ In an analysis of factors that impact geriatric cancer survivors’ quality of life, Pisu et al found that the number of financial hardship events experienced was among the most important predictors of mental health.^36^

Compared with white survivors, black survivors have experienced higher out‐of‐pocket costs,^37^, ^38^, ^39^ a greater number of financial difficulties,^6^ and more financial burden and distress.^38^, ^40^ They were also more likely to file for bankruptcy, experience cancer‐related,^6^ and other medical debt,^34^ to be contacted by a collection agency and to borrow money to pay for medical debt. Not surprisingly, consistent associations have been demonstrated between low household incomes and financial burden among cancer survivors. Low‐income survivors face higher financial distress, and higher financial burden^1^, ^7^, ^20^, ^29^ than higher‐income survivors. They also face higher out‐of‐pocket burdens and are more likely to borrow money to pay for care^9^ and to file for bankruptcy^9^ than survivors with higher incomes.

The literature is less developed in regard to other predictors of financial burden identified in LILAC; however receipt of chemotherapy^31^ and having other chronic conditions in addition to cancer^37^ have been associated with cancer‐related financial problems. Similar to the results of our adjusted models indicating that financial burden was more common among patients diagnosed with regional, but not distant, relative to local stages of disease, Ramsey et al reported that patients who filed for bankruptcy were more likely to have local‐ or regional‐ than distant‐stage disease at diagnosis.^30^ However, it should be noted that the adjusted odds ratios were similar (~1.4) for both regional and distant disease, but relatively few LILAC participants were diagnosed with distant disease resulting in a wider confidence interval that included 1.0. Bankruptcy was extremely rare among LILAC participants and the association we observed between regional (vs local) stage at diagnosis and any financial burden could be driven by associations between stage at diagnosis and other forms of financial burden besides bankruptcy that were included in our measure. Social support has been found to be associated with quality of life in cancer survivors; however less is known about its association with financial burden after cancer.^41^

The LILAC offers a unique source of data to examine financial burdens experienced after cancer. Strengths of this study include its large sample of older women who represent long‐term survivors of 10 different types of cancer from throughout the United States. Potential limitations include the long interval between cancer diagnosis and assessment of financial burden and the possibility of survival and recall bias. Additionally, the LILAC questionnaire asks long‐term cancer survivors whether they experienced any of several types of financial burdens since their cancer diagnosis; however, it is not known when those burdens occurred, and we cannot differentiate between short‐ and long‐term financial impacts. Although women in WHI and LILAC come from across the country and include survivors of several different cancers, they are not representative of the overall population of older women in the United States. Further, although we are unaware of previous work reporting differences in predictors of financial burden by sex, only women are included here and these findings may not generalize to a population of older male survivors.

Cancer‐related financial burden was rare in this population of older female cancer survivors. Predictors of financial burden included both established risk factors, including younger age, African‐American race, and low household incomes, and less‐established factors including comorbidities, chemotherapy, regional stage at diagnosis, and not having private insurance. The identification of several socioeconomic, health‐related and demographic predictors of financial burden may suggest targets of intervention to reduce financial burdens in older cancer survivors.

## AUTHOR CONTRIBUTIONS

Theresa Hastert: conceptualization, writing‐original draft, writing‐review, and editing; Gregory Young and Michael Pennell: conceptualization, formal analysis, writing‐original draft, writing‐review, and editing; Tasleem Padamsee, S. Yousuf Zafar, and Michelle Naughton: conceptualization, writing‐review, and editing; Cecelia DeGraffinreid: conceptualization and project administration; Michael Simon: supervision, writing‐review, and editing; Electra Paskett: conceptualization, funding acquisition, supervision, writing‐review, and editing.

## CONFLICT OF INTEREST

None.
